# Cadmium Pollution Impact on the Bacterial Community Structure of Arable Soil and the Isolation of the Cadmium Resistant Bacteria

**DOI:** 10.3389/fmicb.2021.698834

**Published:** 2021-07-22

**Authors:** Xiaoxia Yu, JinTong Zhao, Xiaoqing Liu, LiXin Sun, Jian Tian, Ningfeng Wu

**Affiliations:** ^1^School of Water Resources and Environmental Engineering, East China University of Technology, Nanchang, China; ^2^Biotechnology Research Institute, Chinese Academy of Agricultural Sciences, Beijing, China

**Keywords:** cadmium pollution, bacterial diversity, seasonal change, Cd-resistant bacteria, bioremediation

## Abstract

Microorganisms play an important role in the remediation of cadmium pollution in the soil and their diversity can be affected by cadmium. In this study, the bacterial community in arable soil samples collected from two near geographical sites, with different degrees of cadmium pollution at three different seasons, were characterized using Illumina MiSeq sequencing. The result showed that cadmium is an important factor to affect the bacterial diversity and the microbial communities in the high cadmium polluted area (the site H) had significant differences compared with low cadmium polluted area (the site L). Especially, higher concentrations of Cd significantly increased the abundance of Proteobacteria and Gemmatimonas whereas decreased the abundance of Nitrospirae. Moreover, 42 Cd-resistant bacteria were isolated from six soil samples and evaluated for potential application in Cd bioremediation. Based on their Cd-MIC [minimum inhibitory concentration (MIC) of Cd^2+^], Cd^2+^ removal rate and 16S rDNA gene sequence analyses, three *Burkholderia* sp. strains (ha-1, hj-2, and ho-3) showed very high tolerance to Cd (5, 5, and 6 mM) and exhibited high Cd^2+^ removal rate (81.78, 79.37, and 63.05%), six *Bacillus* sp. strains (151-5,151-6,151-13, 151-20, and 151-21) showed moderate tolerance to Cd (0.8, 0.4, 0.8, 0.4, 0.6, and 0.4 mM) but high Cd^2+^ removal rate (84.78, 90.14, 82.82, 82.39, 81.79, and 84.17%). Those results indicated that *Burkholderia* sp. belonging to the phylum Proteobacteria and *Bacillus* sp. belonging to the phylum Firmicutes have developed a resistance for cadmium and may play an important role in Cd-contaminated soils. Our study provided baseline data for bacterial communities in cadmium polluted soils and concluded that Cd-resistant bacteria have potential for bioremediation of Cd-contaminated soils.

## Introduction

Heavy metal soil pollution has become a severe environmental problem due to the rapid development of industries such as mining, smelting and agriculture. Moreover, one of the most serious and widespread heavy metal contaminants is cadmium (Cd) (MEP, 2014; Huang et al., 2019). Cd is toxic even at low concentrations of 0.001–0.1 mg L^–1^ and can be accumulated in the human body through the food chain. Once Cd levels become critical, humans can develop emphysema and osteoporosis; and eventually the damage to the lungs, kidneys and liver becomes irreversible. People suffering from severe chronic Cd poisoning develop the itai-itai disease (Satarug et al., 2017; Genchi et al., 2020; Đukić-Ćosić et al., 2020). Consequently, engineering to remedy heavy metal contaminated soil, especially Cd contaminated soil, is urgently needed.

Soil is an important habitat for a diverse group of microorganisms that play an important role in the soil environment. Microorganisms are regarded as sensors of disturbances in the soil ecosystem since they are far more sensitive to environmental stress than macroorganisms (Khan et al., 2010; Yang et al., 2016; Zhang et al., 2016). Moreover, microorganisms in soil are mainly involved in material decomposition, an important process in maintaining soil biological activity as well as regulating soil nutrient circulation. Stable microbial communities mediate soil environment by stabilizing soil structure and maintaining soil physical and chemical conditions (Bissett et al., 2013). Characteristics of microbial community composition and activity were often used as indicators of soil quality (Hamman et al., 2007; Rubin et al., 2013). Many studies have concentered on changes in soil properties owing to the presence of cadmium as it negatively impacts the indigenous microorganism community, impairing the ecological function they provide (Harichová et al., 2012). Therefore, it is indispensable to analyze the response of microbial communities structure in cadmium contaminated soils.

Owing to the difficulty in comprehensively assessing the structure of the microbial communities associated with using traditional techniques, previous research have explored the effects of cadmium contamination on microbial communities using approaches such as denaturing gradient gel electrophoresis (DGGE) (Wang et al., 2006; Zhang et al., 2009), random amplified polymorphic DNA (RAPD) (Wang et al., 2007a), and phospholipid fatty acids (PLFA) (Liao et al., 2010). These approaches, however, only provide limited insights into the bacterial profiles. Recently, the application of high-throughput sequencing based rRNA for evaluating microbial communities is widely used (Luo et al., 2019; Duan et al., 2020).

In addition, microorganisms have large specific surface areas and high metabolic activity, which making them particularly susceptible to the presence of heavy metals in the soil and impeding their ecological function via adsorption, fixation, complexation, dissolution, oxidation reduction, etc. (Vodyanitskii and Plekhanova, 2014). Moreover, a number of soil microbes have been reported effective in the remediation of heavy metals. For example, Suksabye et al. (2016) reported that the addition of *Pseudomonas aeruginosa* or *Bacillus subtilis* to the soil could reduce the amount of Cd in rice grains obtained from Cd contaminated soil as a result of their Cd remediating characteristic. Currently, microbial remediation of heavy metal contaminated soil is regarded a cost-effective, biotechnology approach (Jin et al., 2018). Therefore, it is necessary to isolate Cd-resistant and Cd-adsorbing microorganisms from Cd contaminated soil, which can be used the remediation of Cd polluted soil.

In this study, firstly we explored the microbial community structure of two sites with significantly different Cd levels but of similar geographical location. We also collected samples from both sites during different seasons. High-throughput sequence analysis of 16S rRNA V3-V4 region was used to characterize the microbial taxa and operational taxonomic units (OTUs) distribution in the different samples. This comprehensive analysis enabled us to correlate microbial taxa and OTUs to Cd contamination, thereby illustrating (1) the effects of Cd exposure on microbial community structure in the soil, and (2) the seasonality of microbial activity at the different sites. Secondly, in order to excavate the available microbial resources, 42 Cd-resistant bacteria were isolated and evaluated for potential application in Cd bioremediation.

## Materials and Methods

### Soil Sites and Samples

Soil samples (0–20 cm) were collected from two different arable soil fields located around a mining area in Xiangtan, Hunan Province, China: L site (27°33′N, 113°15′E) and H site (27°46′N, 112°52′E). The distance between the two regions was about 1.3 km. Moreover, three different seasonal samples of both areas were collected: LA and HA in April 2016; LJ and HJ in July 2016; LO and HO in October 2016. Each time three replicates were sampled and stored at −70°C. Samples were divided in three; one part was used for Miseq, the other to determine the Cd concentration and the last one to isolate Cd-resistant bacteria.

### Cd Concentration Analysis

To determine total Cd concentration in the soil samples, 0.5 g air dried sample was digested with 7 mL nitric acid and 3 mL hydrofluoric acid in a polytetrafluoroethylene digestion vessel using a microwave accelerated reaction instrument (CEM-MARS6 Xpress, United States). After complete digestion, the total, water-soluble and filter solution of all samples were measured with a 7,700× Inductively Coupled Plasma Mass Spectrometer (Agilent Technologies, Japan). A heavy metal uncontaminated soil sample (GSS-7, shown in Table 1) was used as a standard negative control.

**TABLE 1:** Total cadmium content of samples.

Samples	Total Cd (mg/kg)	Samples	Total Cd (mg/kg)
LA	0.46 ± 0.023	HA	27.11 ± 3.23
LJ	0.36 ± 0.061	HJ	53.70 ± 0.68
LO	0.39 ± 0.12	HO	8.52 ± 1.01
GSS-7	0.11		

### DNA Extraction and 16S rRNA V3–V4 Region Amplicon Sequencing

Total microbial community DNA of all samples were extracted following the Mobio Power Soil DNA isolation Kit protocols. V3-V4 regions of 16S rRNA were amplified using the primers 338F (5′-ACTCCTACGGGAGGCAGCAG-3′) and 806R (5′-GGACTACHVGGGTWTCTAAT-3′). Sequencing of the amplicons was outsourced to Allwegene (Beijing, China); amplicons were sequenced on an Illumina Miseq platform. All the sequences data have been submitted to NCBI (National Center for Biotechnology Information), accession numbers were: SAMN07643022 (HA1), SAMN07644976 (HA2), SAMN07644984 (HA3); SAMN07644992 (HJ1), SAMN07644993 (HJ2), SAMN07644994 (HJ3); SAMN07644995 (HO1), SAMN07644997 (HO2), SAMN07645000 (HO3); SAMN07645001 (LA1), SAMN07645003 (LA2), SAMN07645004 (LA3); SAMN07645005 (LJ1), SAMN07645006 (LJ2), SAMN07645007 (LJ3); SAMN07645009 (LO1), SAMN07645010 (LO2), and SAMN07645011 (LO3).

### Sequence Analysis

Sequence analysis of the 16S rRNA V3-V4 region amplicon sequences was performed using QIIME Pipeline, Version 1.8.0 (Caporaso et al., 2010). Firstly, low quality reads (average quality score <20) were trimmed and paired-ends sequences were merged to be single sequence according to their overlap sequence (>10 bp). Then, sequences from the samples were distinguished on the basis of the barcodes and primers. Finally, chimeras were deleted using USEARCH (Edgar et al., 2011) and the smaller sequences were removed by MOTHER (Schloss et al., 2009). These high quality sequences were subsequently used for downstream analysis. OTUs were clustered using a 97% identity threshold using UCLUST v1.2.22 (Edgar, 2010) and representative OUT sequence was obtained. Moreover, singleton OUT was removed. A total of 338,077 final sequences were gained for all the samples after filtering for low quality sequences. OTUs were annotated using the Ribosomal Database Project classifier (Wang et al., 2007b). Alpha-diversity (Chao 1, observed species, goods coverage and Shannon) and beta-diversity [principal component analysis (PCA) and Metastats] were analyzed based on Miseq sequence data.

### Isolation and Identification of Cd-Resistant Bacteria

To isolate Cd-resistant bacteria, soil samples (2 g) were placed in sterile 0.9% NaCl (18 mL) at 30°C and shaken at 200 rpm for 0.5 h to completely separate bacteria from soil. After settling for several minutes, an aliquot of the suspension was serially diluted (from 10^–1^ and 10^–4^). Each diluted solution was spread onto an Burk agar plates (0.8 g/L KH_2_PO_4_, 0.262 g/L K_2_HPO_4_⋅3H_2_O, 1 g/L (NH_4_)_2_SO_4_, 0.2 g/L MgSO_4_⋅3H_2_O, 1 g/L yeast extract, and 1.5% agar) containing progressively higher concentrations of CdCl_2_ (1, 3, and 5 mM). To isolate Cd-resistant and bio-safe *Bacillus* bacteria, the bacterial enrichment cultures were heat-shocked at 80°C for 20 min and aerobic *Bacillus* sp. were isolated from the soil by plating on LB agar plates containing different concentrations of CdCl_2_ (0, 0.5, 1, and 2 mM). Bacterial growth was observed after incubation at 30°C for 24 h. Single colony was picked with sterilized wire loop and re-streaked on CdCl_2_ supplemented LB agar plates and again incubated at 30°C for 24 h. The process was repeated until the pure culture was obtained. Genomic DNA was isolated using the TIANamp Bacteria DNA kit (TIANGEN Biotech). The 16S rRNA gene was amplified from the extracted DNA using the universal primers the universal forward primer 27f (5′-AGAGTTTGATCCTGGCTCAG-3′) and reverse primer 1492r (5′-TACGGTTACCTTGTTACGACTT-3′). The amplification products were cloned in the pGM-T (TIANGEN Biotech) vector using competent *Escherichia coli* TOP10 cells (TIANGEN Biotech). Sequencing was carried out using T7 and SP6 primers and compared to the GenBank database using the NCBI BLAST program.

### Evaluation of Cadmium Resistance and Determination of Cadmium Removal Rate

To evaluate growth in a liquid medium of isolated bacteria, the MIC of Cd^2+^ (MIC-Cd) was determined. LB medium (800 μL) with different concentrations of Cd^2+^ was dispensed into 96-well (12 × 8) microtiter plates (96 × 2-mL wells) with a multi-channel micropipette (rows A to H: 0, 1, 2, 3, 4, 5, 6, and 7 mM). Single colonies of the test strains were inoculated into 3 mL of LB medium and cultured overnight. The test culture (15 μL) was then inoculated into each well of the prepared 96-well plate. After 24 h at 30°C and 750 rpm in an incubator (Heidolph, Viertrieb, Germany), 200 μL of the cell suspension was transferred to a 96-well plate and the turbidity at OD_600_ was measured.

To determine the Cd^2+^ adsorption of isolated bacteria, growth of cells was grown in LB liquid medium supplementation with 0.1 mM CdCl_2_ and shaken at 200 rpm at 30°C for 24 h. Cells were harvested by centrifugation at 12,000 rpm for 10 min and the supernatant then diluted to an appropriate concentration for analysis. Cd^2+^ concentrations in culture supernatants were measured via atomic absorption spectrophotometry (Z-2000, Hitachi, Japan), with Cd^2+^ removal rate being calculated using the following equation:

Removal rate (%) = (*C*_*i*_ −*C*_*e*_)/*C*_*i*_ × 100

where *C*_*i*_ and *C*_*e*_ are the initial and equilibrium Cd^2+^ concentrations (mM), respectively.

## Results

### Cd Concentration of Different Samples

Cd concentrations in all the samples were shown in Table 1. Cd concentrations were significantly higher in samples collected from site H (8.52–53.70 mg kg^–1^) as compared to site L (0.36–0.46 mg kg^–1^). Among six samples, the Cd concentrations of HJ (53.70 mg kg^–1^) was highest while the Cd concentrations of LJ was lowest (0.36 mg kg^–1^). Moreover, Cd concentrations of soil samples from site L were similar across the different seasons, whereas there was an obvious seasonal effect on the Cd concentrations for the H site samples. Specifically, the Cd content of HJ was about 2.0-fold and 6.3-fold higher than that of HA and HO, respectively.

### Effects of Cd Concentration and Seasons on Soil Microbial Community Structure

**FIGURE 1:**
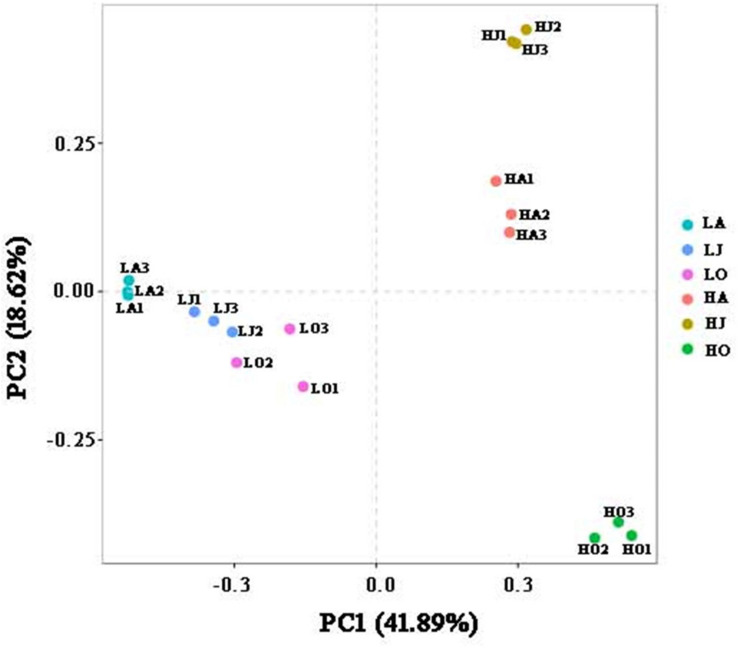
Principal component analysis (PCA) displaying beta-diversity of microbial diversity between samples. PCA was calculated using weighted Unifrac distance. A total of 41.89 and 18.62% of the variations in the bacterial communities could be explained by the first and second principal components, respectively.

Rarefaction curve analysis showed that the quantity of OTUs was enough to reach saturation, indicated that the sequencing depth was sufficient to characterize the microbial community composition (Supplementary Figure 1). The OTU densities of soil samples from site H increased with increasing Cd concentration (HJ > HA > HO), while the OTU density of sample LJ with the lower Cd contamination level was higher than those of sample LA and LO (Supplementary Table 1). Comparison of the different sites in the same season showed that HA and HJ have more defined OTUs than LA and LJ, respectively. Bacterial alpha diversity, including the chao1, observed species, PD whole tree and Shannon, varied among the six soil samples. We observed the highest diversity at location with HJ, whereas the lowest diversity at location with HO (Supplementary Figure 2). However, results of the PCA supported the previous results, showing that soil microbial communities clustered strongly based on Cd concentration grade (Figure 1). A total of 41.89 and 18.62% of the variations in the bacterial communities could be explained by the first and second principal components, which also indicated that the cadmium concentration is the key factor to affect the bacterial diversity of the soil (Figure 1). Further, all samples from site L (LA, LJ, and LO) clustered tightly whereas samples from site H were more dispersed (Figure 1). These results indicated that the seasonal change in microbial community structure in high Cd contaminated soil was more distinct than in low Cd contaminated soil.

### Effects of Cd Concentration and Seasons on Soil Microbial Community Composition and Diversity

**FIGURE 2:**
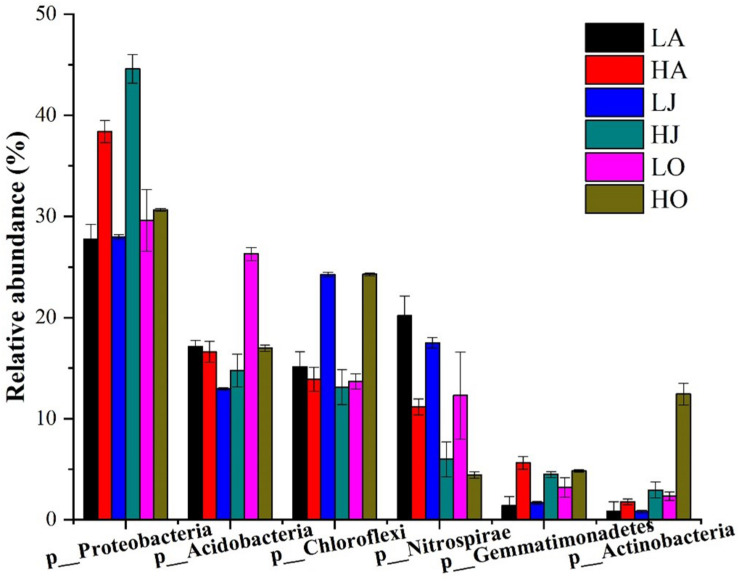
Community composition of dominant bacterial phyla for six samples of different Cd concentrations (LA: 0.46 mg/kg; LJ: 0.36 mg/kg; LO: 0.39 mg/kg; HA: 27.11 mg/kg; HJ: 53.70 mg/kg; HO: 8.52 mg/kg). The relative abundances of Proteobacteria (HA:38.4%, LA:27.77%; HJ:44.59%, LJ:27.98%; HO:30.65%, and LO:29.60%) and Gemmatimonadetes (HA:5.62%, LA: 1.41%; HJ:4.48%, LJ:1.69%; HO:4.82%, and LO:3.21%) at the site H were higher than that of site L, whereas the abundance of Nitrospirae (HA: 11.16%, LA: 20.17%; HJ: 5.98%, LJ: 17.49%; HO: 4.43%, and LO: 12.28%) at the site H was lower than that of site L.

A total of 338,077 high-quality bacterial 16S rRNA gene sequences were obtained from 18 samples, ranging from 10,812 to 23,059 sequences per sample (Supplementary Table 1). Among the total 16S rRNA gene sequences, 71 sequences were classified as archaea, accounting for 0.02% of total sequences. The remaining sequences belonged to bacteria (338006 of 338077), accounting for 99.98% of the 16S rRNA gene sequences, and presented 47 phyla. Specifically, the dominant phyla, consisting of 1% (relative abundance) or more to total community composition, are shown in Figure 2. Proteobacteria (27.77–44.59%), Acidobacteria (12.95–26.29%), Chloroflexi (13.11–24.28%), Nitrospirae (4.43–20.17%), Gemmatimonadetes (1.40–5.62%), and Actinobacteria (0.80–12.43%) were the six largest phyla in all samples. Among these more abundant phyla, it was observed that the relative abundances of Proteobacteria, Gemmatimonadetes, and Actinobacteria at the site H were higher than that of site L, whereas the abundance of Nitrospirae at the site H was lower than that of site L (Figure 2). Additionally, we found that the relative abundances of five levels (phylum, class, order, family, and genus) of *Gemmatimonas* at the site H were all higher than that of site L. However, the relative abundances of four levels (phylum, class, order, and family) of *Nitrospira* at the site H were all lower than that of site L while the abundances of *Nitrospira* at the site H was higher than that of site L (Supplementary Figure 3).

**FIGURE 3:**
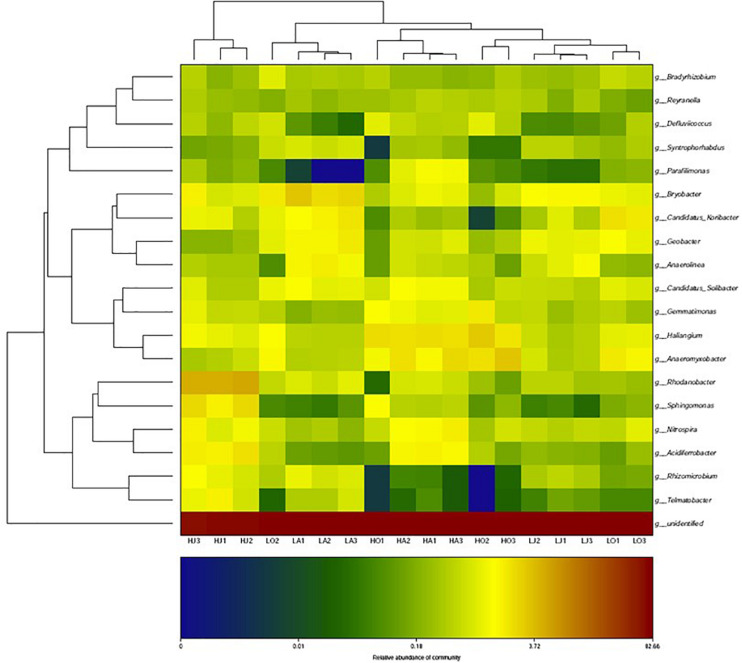
Heatmap of the cluster analysis for top-20 most abundant bacteria at genus-level for the six samples of different Cd concentrations (LA: 0.46 mg/kg; LJ: 0.36 mg/kg; LO: 0.39 mg/kg; HA: 27.11 mg/kg; HJ: 53.70 mg/kg; HO: 8.52 mg/kg).

Figure 3 shows a heatmap of soil bacterial community for six samples of different Cd content at the genus level. From the corresponding cluster analysis of the 20 abundant bacterial genera, the *Bryobacter*, *Geobacter*, *Gemmatimonas*, *Haliangium*, *Anaeromyxobacter*, *Nitrospira*, *Candidatus of Koribacter*, and *Candidatus of Solibacter* had higher contents in most soil samples. Among these more abundant bacterial genera, it was observed that the relative abundances of *Gemmatimonas, Haliangium*, and *Anaeromyxobacter* at the site H were all higher than that of site L, whereas the abundance of *Bryobacter*, *Geobacter*, and *Candidatus of Koribacter* at site H were all lower than that of site L. Specifically, the abundance of *Rhodanobacter*, *Sphingomonas*, *Nitrospira*, *Acidiferrobacter*, *Rhizomicrobium*, and *Telmatobacter* at the site HJ were significantly higher than that of LJ (Figure 3).

Further, relative abundances of dominant phyla and genera were influenced by the season. For Proteobacteria, relative abundance (LA: 27.77%, LJ: 27.98%, and LO: 29.60%) were similar in the different seasons at site L, but very different at site H (HA: 38.4%, HJ: 44.59%, and HO: 30.65%). Contrastingly, Acidobacteria abundance at site L (LA: 17.12%, LJ: 12.95%, and LO: 26.29%) were strongly dependent on the season in which the soil sample was taken, whereas seasonality did not greatly affect its abundance at site H (HA: 16.60%, HJ: 14.77%, and HO: 16.95%). In the case of *Nitrospira* abundance, seasonal changes were observed at both sites (LA: 20.17%, LJ: 17.49%, and LO: 12.28%; HA: 11.16%, HJ: 5.98%, and HO: 4.43%) (Table 2). Additionally, the relative abundances of the 20 abundant bacterial genera at site H and L were different in varying seasons (Figure 3). Taken together, these data clearly suggest that seasonal conditions impact the soil microbiome and the abundance of susceptible populations during seasons responded to the soil Cd content.

**TABLE 2:** Pearson correlation and t-test analysis of the relative abundance of microbial phyla, classes, and cadmium content.

	LA	LJ	LO	HA	HJ	HO	*r*^*e*^	*p*^*f*^
Cd (mg/kg)	0.460	0.360	0.390	27.110	53.700	8.520		
p^*a*^__Proteobacteria (%)	27.770	27.975	29.605	38.396	44.592	30.652	0.989	0.087
c^*b*^__Alphaproteobacteria (%)	5.561	4.368	6.017	7.145	12.458	7.806	0.921	0.108
c__Betaproteobacteria (%)	5.776	4.882	6.027	7.823	9.649	4.173	0.892	0.240
c__Gammaproteobacteria (%)	3.476	2.733	2.748	4.868	11.253	2.109	0.941	0.191
c__Deltaproteobacteria (%)	12.855	15.873	14.740	18.485	11.190	16.542	−0.329	0.395
p__Acidobacteria (%)	17.121	12.947	26.287	16.597	14.766	16.951	−0.340	0.256
p__Chloroflexi (%)	15.130	24.264	13.691	13.902	13.114	24.276	−0.460	0.466
p__Nitrospirae (%)	20.174	17.488	12.281	11.166	5.980	4.433	−0.596	0.006
c__Nitrospira (%)	20.174	17.488	12.281	11.166	5.980	4.433	−0.596	0.006
o__Nitrospirales (%)	20.174	17.488	12.281	11.166	5.980	4.433	−0.596	0.006
f__Nitrospiraceae (%)	15.048	11.798	6.538	6.868	4.521	1.801	−0.468	0.011
p__Gemmatimonadetes (%)	1.408	1.687	3.209	5.624	4.476	4.819	0.623	0.031
c__Gemmatimonadetes (%)	1.408	1.687	3.209	5.624	4.476	4.819	0.623	0.031
o^*c*^__Gemmatimonadales (%)	1.084	1.387	2.920	5.165	4.342	4.711	0.635	0.023
f^*d*^__Gemmatimonadaceae (%)	1.084	1.387	2.920	5.165	4.342	4.711	0.635	0.023
p__Actinobacteria (%)	0.854	0.800	2.342	1.777	2.942	12.433	−0.011	0.133
p__Saccharibacteria (%)	0.022	0.006	0.016	0.131	0.675	0.247	0.910	0.093
p__Cyanobacteria (%)	0.474	0.394	0.428	0.173	1.306	0.150	0.712	0.404
p__Chlamydiae (%)	0.404	0.356	0.117	0.207	0.760	0.111	0.691	0.371
p__Bacteroidetes (%)	2.485	1.478	1.321	2.933	3.255	0.368	0.681	0.322
p__Spirochaetae	0.630	0.469	0.438	0.178	0.100	0.038	−0.659	0.002

*P^*a*^ represents phylum, C^*b*^ represents class, o^*c*^ represents order, f^*d*^ represents family, *r*^*e*^ represents the Pearson correlation coefficient between the bacterial relative abundance and Cd concentration in soil, *p*^*f*^ represent the *p*-value of the bacterial relative abundance difference between the H and L samples in *t*-test analysis.*

To further assess the relative influence of total Cd concentration on microbial taxa, the Pearson correlations between the relative abundant phyla and Cd were calculated (Table 2). The results showed that the relative abundances of Proteobacteria (*r* = 0.989), Gemmatimonas (*r* = 0.623), Saccharibacteria (*r* = 0.910), Cyanobacteria (*r* = 0.712), Chlamydiae (*r* = 0.691), and Bacteroidetes (*r* = 0.681) all positively correlated with Cd concentration, while the abundance of Nitrospirae (*r* = −0.596) and Spirochaetae (*r* = −0.659) correlated negatively with Cd concentration (Table 2). Of the Proteobacteria, the relative abundances of Alphaproteobacteria, Betaproteobacteria, and Gammaproteobacteria were all found to be positively correlated with Cd concentration with the exception of Deltaproteobacteria (Table 2). In agreement, the phylum Proteobacteria has previously been reported to be associated with Cd.

### Isolation of Cd-Resistant Bacteria and Their Potential for Reducing Cd Concentration

**FIGURE 4:**
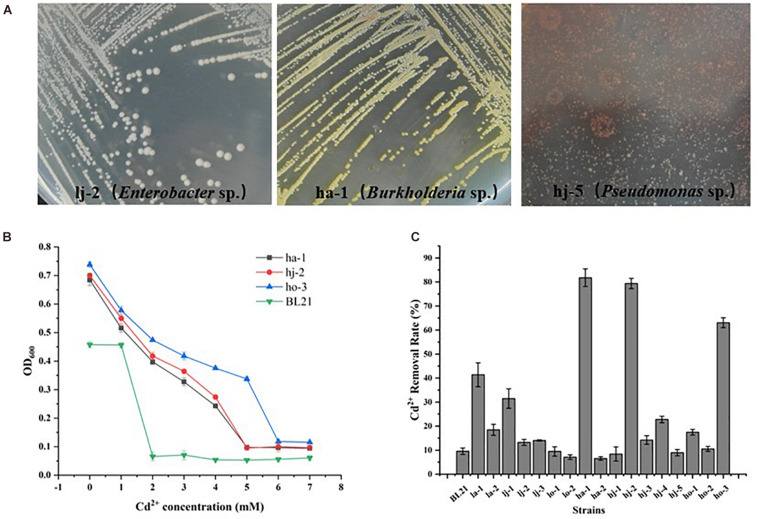
Colony morphology of Cd-resistant bacteria and evaluation of their minimum inhibitory concentration of Cd^2+^ (Cd-MIC) and Cd^2+^ removal rate. **(A)** Colony morphology of partial Cd-resistant strains. **(B)** Determination of Cd-MIC for Cd-resistant bacteria in varied at different concentrations of Cd. **(C)** Evaluation of Cd^2+^ removal rate for Cd-resistant bacteria, which was measured in LB liquid medium supplementation with 0.1 mM CdCl_2_ and shaken at 200 rpm at 37°C for 24 h.

Once having a basic understanding of the bacterial community response to Cd pollution, the potential of microbial bioremediation to reduce Cd levels was investigated. Firstly, the Cd-resistant bacteria were isolated. A total of 17 strains of different morphological bacteria (Colony morphology of partial Cd-resistant strains were showed in Figure 4A) were isolated from six Cd-contaminated samples using Bulk agar plates containing 1, 2, and 3 mM Cd^2+^. Then the MIC-Cd values and the Cd^2+^ removal efficiency of those isolates and 1 negative control strains (*E. coil* BL21) were determined (Supplementary Table 2). Among them, the strains ho-3, ha-1, and hj-2 exhibited the higher level of Cd^2+^ resistance than other strains. In detail, the Cd-MIC value of ho-3, ha-1, and hj-2 was 6, 5, and 5 mM, respectively, markedly higher than that of the control strain *E. coli* BL21 (Cd-MIC: 2 mM Cd^2+^) (Figure 4B). However, as shown in Figure 4C, ha-1 exhibited the highest Cd^2+^ removal efficiency (81.78%), which was higher than that observed for ho-3 (63.05%) and hj-2 (79.37%), whilst the cadmium removal efficiency of *E. coli* BL21 was 9.57%. These results indicate that Cd-resistant bacteria have potential for bioremediation of Cd-contaminated soils. In addition, all of the 17 bacteria were identified through 16S rDNA analysis (Supplementary Table 2). Interestingly, ha-1, hj-2, and ho-3 were identified as *Burkholderia sp.*, which belong to the class of Betaproteobacteria, phylum Proteobacteria. Those results further emphasized the importance of Proteobacteria in Cd pollution remediation. However, the genus *Burkholderia* contains large number of diverse species which include many phytopathogens, such that a group of 17 closely related *Burkholderia* species, the *Burkholderiacepacia* complex (BCC), are responsible for prevalent and potentially lethal pulmonary infections in immunocompromised individuals, such as individuals with cystic (Elshafie and Camele, 2021). Consequently, both Cd resistant and biosafety strains were further screened.

### Isolation of *Bacillus* sp. Bacteria With Cd-Resistance From Samples HJ and LJ and Evaluation of Their Cd^2+^ Removal Rate

*Bacillus* sp. are well-known biocontrol agents against various fungal plant pathogens (Jangir et al., 2018). To isolate Cd-resistant and bio-safe bacteria, *Bacillus* sp. were isolated from the soil samples. According to the above results, there were significant differences in Cd content (HJ: 53.70 mg kg^–1^, LJ: 0.36 mg kg^–1^) and microbial community structure between soil samples HJ and LJ (OTUs no. of HJ: 2093, OTUs no. of LJ:1843) although these two soil samples were collected at the same season (Table 1 and Supplementary Table 1). Thus, we focused on screening *Bacillus* sp. with Cd-resistance from HJ and LJ samples. Supplementary Figure 4 showed that the observation of the screening Cd-resistant *Bacillus* sp. strains on the LB agar containing 1mM Cd^2+^ after incubation at 30°C for 24 h. For HJ, more than 100 colonies were grown on LB agar, whilst about 15 large colonies were screened for LJ in same conditions (Supplementary Figure 4). Additionally, the diversity of microbial morphology in HJ was significantly higher than that in LJ (Supplementary Figure 4). Those results were accordance with the microbial community diversity of samples HJ and LJ.

**FIGURE 5:**
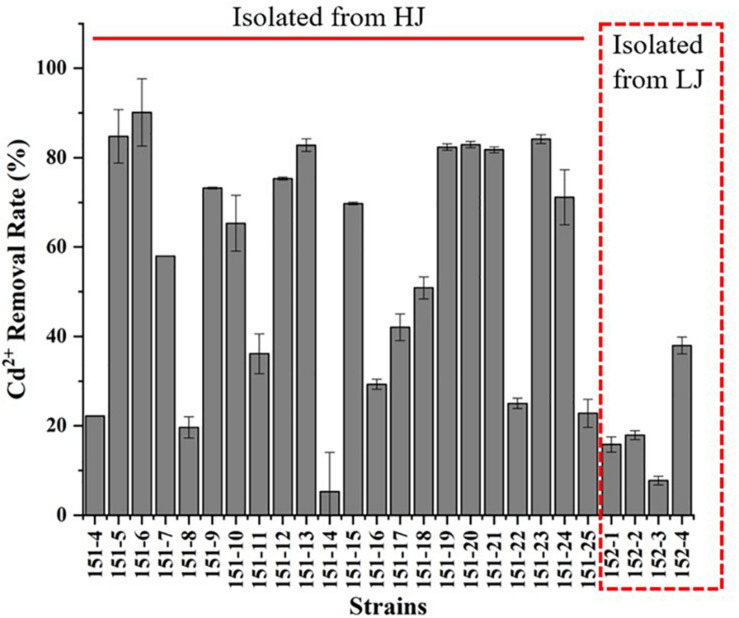
Evaluation of Cd^2+^ removal rate for Cd-resistant *Bacillus* sp. bacteria, which was measured in LB liquid medium supplementation with 0.1 mM CdCl_2_ and shaken at 200 rpm at 37°C for 24 h.

Finally, a total of 21 strains were isolated from HJ while only 4 strains were obtained from LJ. All strains were identified as *Bacillus* sp. (Supplementary Table 3). And the Cd-MIC and Cd^2+^ removal rate of those 25 strains were showed in Supplementary Table 3 and Figure 5. The result showed that *Bacillus* sp. strains 151-6 isolated from HJ exhibited the highest Cd^2+^ removal efficiency (90.14%), which was higher than that observed for ha-1 (81.78%). Except for 151-6, the Cd^2+^ removal rate of other five *Bacillus* sp. strains isolated from HJ were more than 80% (151-5: 84.78%, 151-13: 82.39%, 151-21: 81.79%, and 151-23: 84.18%). However, the Cd^2+^ removal rate of 4 *Bacillus* sp. strains (named as 152-1, 152-2, 152-3, and 152-4) obtained from LJ was 15.84, 17.92, 7.74, and 37.94%, respectively. Additionally, the Cd-MIC of *Bacillus* sp. strains were lower than that of *Burkholderia* sp. strains while some of the *Bacillus* sp. strains exhibited higher Cd^2+^ removal rate. Those results indicated that *Bacillus* sp. strains may have a great potential in remediation of cadmium contamination.

## Discussion

The function of soil ecosystem depends heavily on soil microbes as they can promote material circulation, nutrient transformation, energy flow, organic matter decomposition and other ecosystem-related biochemical processes (Lozupone et al., 2007; Luo et al., 2019). Many studies use soil microbial communities to evaluate the ecological status of heavy metal contaminated soils (Stefanowicz et al., 2010; Gómez-Sagasti et al., 2012; Burges et al., 2015). Detecting changes in microbial populations and activities is generally more feasible than directly evaluating the physicochemical properties of soil, as the identification of soil microbes may contribute to the evaluation of remediation treatment. The application of high-throughput sequencing based rRNA is more accurate for evaluating soil microbial communities (Luo et al., 2019).

Previous studies have reported that the presence of heavy metals can influence the microbial diversity and also change the community structure and function (Luo et al., 2019; Duan et al., 2020). Wang et al. (2006) found that low contend of heavy metals can stimulate the growth of microorganisms, while at greater concentrations, an inhibitory effect becomes more prominent, which often leads negatively correlated outcomes. Hence, the concentration of Cd in the soil is critical. In this study, we found that there was an obvious seasonal effect on the Cd concentrations for the H site samples. It may be caused by the change of soil basic parameters in different seasons, such as precipitations, temperature, oxygen, pH and so on (Zhang et al., 2007; Yang et al., 2016; Zoghlami et al., 2018). Previous study has reported that the soil Cd content around rice roots was also greatly varied with growing seasons in Hunan Province, China, and the most important factors were humidity and temperature (Liu et al., 2017). Soil Cd concentration in Apr. (HA) might be more likely to subside to deep soil due to high humidity. Whilst soil Cd content in Jul. (HJ) was significant increase might since soluble Cd^2+^ was moved from deep soil to surface layer because of evaporation. On the contrary, soil Cd content in Oct. (HO) was subsided again might be because of low temperature and soil moisture transform. However, the Cd content of soil samples from site L were similar across the different seasons, probably as the Cd concentration in the soil was relatively low and could not cause significant change.

There is considerable debate about the effects of Cd on microbial growth. Previous study reports that Cd addition (>1 mg kg^–1^) of red paddy soil can inhibit soil microbial biomass (Guo et al., 2018). However, as to yellow cinnamon soils under *Xanthoceras sorbifolium* Bunge and forest soils of Haplic Cambisols in Northeast China, the opposite result was found (Jiang et al., 2018; Duan et al., 2020). Hence, the effects of Cd on microorganism are closely related to the concentrations of Cd and soil type. In our study we found that a significant increase in bacterial diversity with increasing Cd concentrations in site H (Supplementary Table 1 and Supplementary Figure 2), where the soil type is yellow. The result is consistent with the study on yellow cinnamon soils, who found that for all Cd groups (10–100 mg kg^–1^), 100 mg kg^–1^ of Cd treatment soil bacterial diversity of yellow cinnamon soils under *X. sorbifolium* Bunge was higher than that of 10 mg kg^–1^.

One previous study also reported a variation in bacterial community response to Cd contamination of agricultural paddy soil (Luo et al., 2019). Our results also illustrate changes in the bacterial community composition caused by the Cd polluted (Figures 2, 3, Supplementary Figure 3, and Table 2). This is probably as the community readjustment in response to the introduction of Cd, decreased the number of metal-sensitive microorganisms and increased the number of resistant microorganisms in the soil, which eventually led to changes in community composition. The adaption mechanism of these bacterial populations may be attributed to different microorganism life activities. In our study, the relative abundance of Proteobacteria (*r* = 0.989) and Gemmatimonas (*r* = 0.623) were all positively correlated with Cd concentration, while the abundance of Nitrospirae (*r* = −0.596) and Spirochaetae (*r* = −0.659) correlated negatively with Cd concentration (Table 2), suggesting that Proteobacteria and Gemmatimonas are Cd-tolerant, whereas Nitrospirae and Spirochaetae are Cd-sensitive. In agreement, the phylum Proteobacteria has previously been reported to be associated with Cd. A large number of isolated and identified strains that resisted Cd or absorbed Cd belong to Proteobacteria and include *Burkholderia* (Wang et al., 2020), *Sphingomonas* (Cheng et al., 2021), *Pseudomona* (Chellaiah, 2018), and *Rhizobium* (Li et al., 2019). Also many Gram-negative bacteria belonging to Proteobacteria such as *Acinetobacter*, *Ralstonia* (also named *Cupriavidus*) and *Comamonas* exclusively exist in a Cd-cultivation library of mine tailing (Zhang et al., 2007). Moreover, the result of isolating Cd-resistant bacteria showed that three *Burkholderia* sp. (ha-1, hj-2, and ho-3) exhibited strong Cd-tolerance and high Cd^2+^ removal rate. Our study confirms that Proteobacteria may have developed a resistance for Cd and play an important role in Cd-contaminated soils. As for Gemmatimonas, the relative abundances of five levels (phylum, class, order, family, and genus) of *Gemmatimonas* at the site H were all higher than that of site L, suggesting that *Gemmatimonas* are Cd-resistant. The resistance mechanism is related to the precipitation-dissolution balance, which limits the dynamic changes of free metal ions in the soil (Abbas et al., 2018). *Gemmatimonas aurantiaca*, which makes up about 2% of soil bacterial communities, has been reported to accumulate polyphosphate (Zhang et al., 2003). Moreover, the accumulation of polyphosphate has been linked with heavy metal tolerance in bacteria, yeasts and fungi (Trilisenko et al., 2017; Kulakovskaya, 2018; Sathendra et al., 2018; Kolhe et al., 2020). Therefore, it is possible that *Gemmatimonas* can accumulate polyphosphate and precipitate of Cd^2+^ in the soil and therefore explains the higher abundance of this phylum at site H. For microbial communities, heavy metal-resistance species can compensate for the loss of metal-sensitive species, ensuring a stable microecological environment (Awasthi et al., 2014). Nitrospirae, a major bacteria group in our soil samples, possesses a higher heavy metal sensitivity, as reported by previous study (Luo et al., 2019). We found that the relative abundances of four levels (phylum, class, order, and family) of *Nitrospira* at the site H were all lower than that of site L while the abundances of *Nitrospira* at the site H was higher than that of site L (Supplementary Figure 3). Nitrospira are nitrite-oxidizing bacteria (Han et al., 2017) and generally Cd can inhibit nitrification efficiency (Li et al., 2015; Wang et al., 2016), the highly Cd contaminated soils at site H were believed to inhibit growth of Nitrospira. Whereas the result of the abundances of *Nitrospira* with response of Cd was opposite. We inferred that the genus annotation may not be completely.

Identifying key heavy metal-resistant bacterial under Cd stress is very important for remediation of Cd-contaminated soils. In this study, we found that two *Bacillus* sp. strains, 151-6 (Cd^2+^ removal rate: 91.22%, Cd-MIC: 0.4 mM) and 151-25 (Cd^2+^ removal rate: 22.80%, Cd-MIC: 1.0 mM), isolated from the same soil sample but exhibit significant differences in Cd^2+^ resistance and Cd^2+^adsorption. Our previous work has elucidated the mechanism of Cd-resistance of 151-6 and 151-25, a cadmium efflux system accessory protein and a cadmium resistance protein, was found to play a major role on the Cd^–^ resistance (Yu et al., 2020). Future, the mechanism of Cd-adsorption of 151-6 and 151-25 will be explored. Of course, we also paid attention to the relative abundance of *Bacillus* sp. in soil samples HJ and LJ, which was 0.050 and 0.017%, respectively. This data is consistent with isolation result of *Bacillus* sp. bacteria from samples HJ and LJ. The result indicated that *Bacillus* sp. strains may have a great potential in remediation of cadmium contamination.

Finally, many studies have reported that microbial communities can influenced by specific soil physicochemical properties, such as pH, organic matter, available phosphorus, hydrolytic nitrogen and so on. Unfortunately, we overlooked the effect of these factors on microbial community and the concentration of Cd at the beginning of our experiment. Thus, the basic physiochemical parameters of the six samples were not obtained. However, we recollected the soil samples of the site H and L in June 2021, and the pH and organic matter were measured. The soil pH of the site H and L was 5.04 and 6.56, respectively. And the organic matter of the site H and L was 60.14 and 36.58 g/kg, respectively. The pH value is the key determinant affecting the solubility and liquidity of metal ions, and heavy metal mobility and bioavailability increase due to competition for ligand between H^+^ ions and dissolved metals. Organic matter can enhance the accumulation of organic carbon in the soil, thus increasing the adsorption of Cd^2+^ in the soil. It is likely that the dynamic changes of microbial community structure, cause by heavy metals, may be closely related to the type and chemical morphology of metals and soil physicochemical properties.

In conclusion, we characterized the diversity of the bacterial community in two different Cd contaminated soils collected in three different seasons by high throughput Illumina MiSeq sequencing. The result showed that long-term Cd pollution and season change could cause remarkable changes in bacterial population abundance and composition structure. Then, to excavate the available microbial resources, 42 Cd-resistant bacteria were isolated and evaluated for potential application in Cd bioremediation. Our results showed that both selected *Burkholderia* sp. and *Bacillus* sp. strains have potential for bioremediation of Cd-contaminated soils. Therefore, our study provided baseline data for bacterial communities in cadmium polluted soils and concluded that Cd-resistant bacteria have potential for bioremediation of Cd-contaminated soils.

## Data Availability Statement

The datasets presented in this study can be found in online repositories. The names of the repository/repositories and accession number(s) can be found in the article/Supplementary Material.

## Author Contributions

XY, JZ, JT, and NW conceived and coordinated the study and wrote the manuscript. XY, JZ, and JT designed, performed, and analyzed the experiments. XL and LS provided technical assistance and contributed to the preparation of the figures. All authors reviewed the results and approved the final version of the manuscript.

## Conflict of Interest

The authors declare that the research was conducted in the absence of any commercial or financial relationships that could be construed as a potential conflict of interest.
