# Supplementary material for: Cadmium Pollution Impact on the Bacterial Community Structure of Arable Soil and the Isolation of the Cadmium Resistant Bacteria
Source: Front Microbiol. 2021 Jul 22;12:698834. doi: 10.3389/fmicb.2021.698834 (PMC8339475; doi:10.3389/fmicb.2021.698834)
Supplement: Supplementary file 1 [file Table_1.DOCX]

Supplementary Material

**
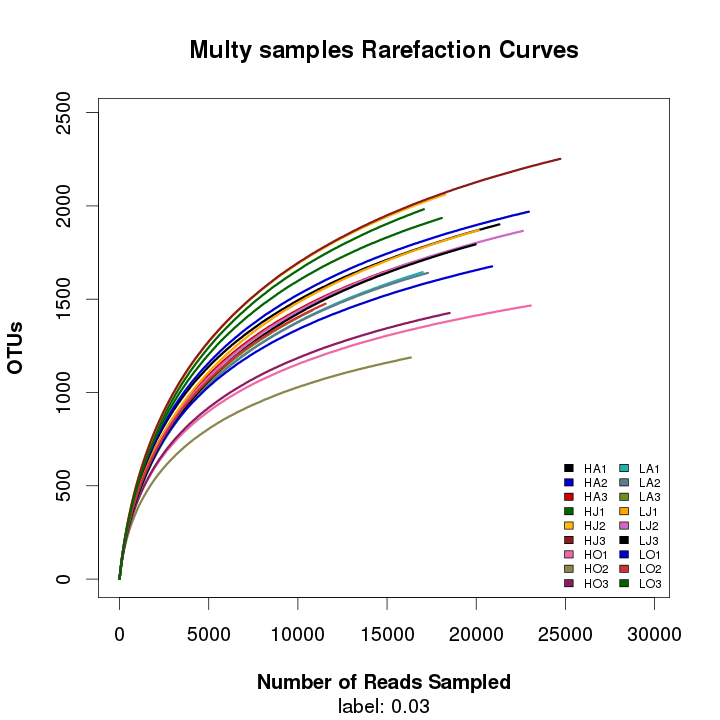
**

**Fig. S1** Rarefaction curve of all samples. Each sample has three biological replicates.

**
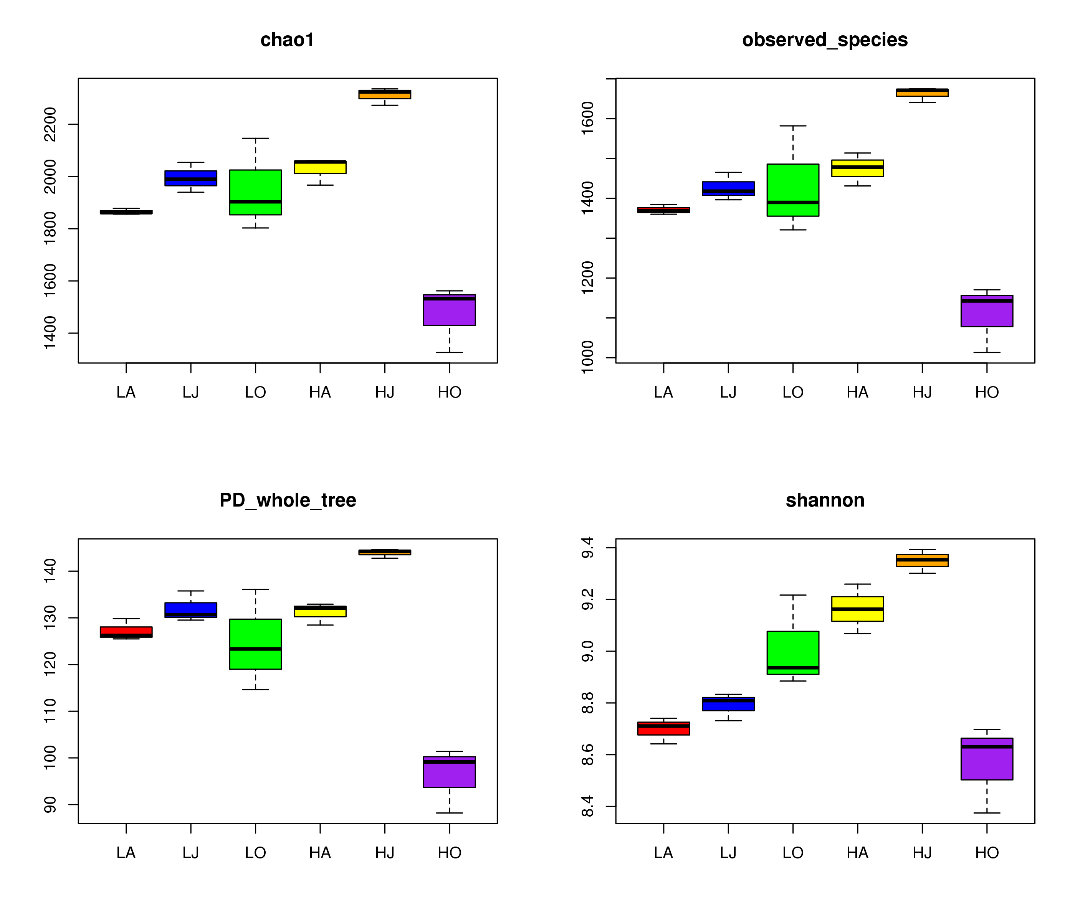
**

**Fig. S2** Soil bacterial abundance and diversity indexes under different concentrations of Cd (LA: 0.46 mg/kg; LJ: 0.36 mg/kg; LO: 0.39 mg/kg; HA: 27.11 mg/kg; HJ: 53.70 mg/kg; HO: 8.52 mg/kg)


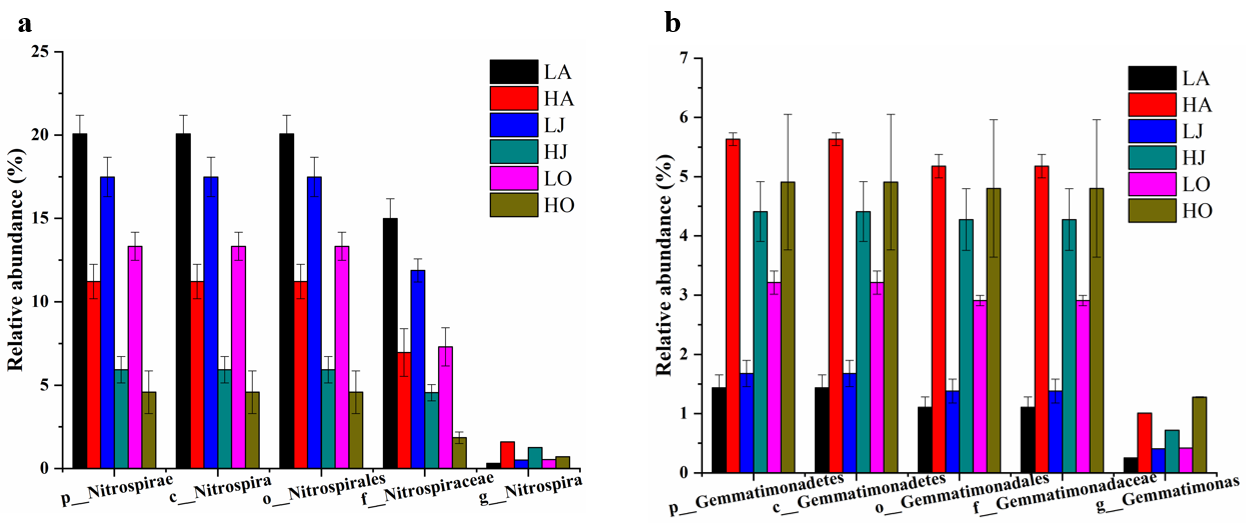


**Fig. S3** Comparison of different levels of *Nitrospira* and *Gemmatimonas* in Cd contaminated soil samples. (a) Comparison of different levels of *Nitrospira* in Cd contaminated soil samples; (b) Comparison of different levels of *Gemmatimonas* in Cd contaminated soil samples.


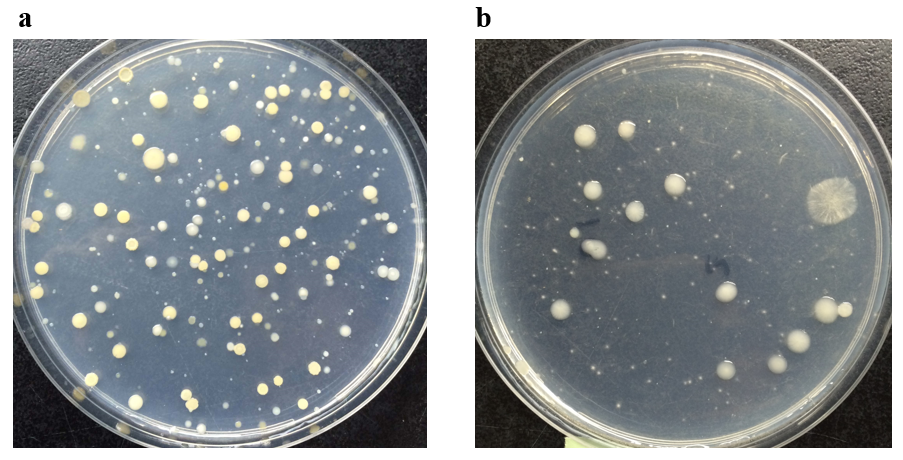


**Fig. S4** The observation of the screening Cd-resistant *Bacillus* sp. strains on the LB agar containing 1mM Cd^2+^ after incubation at 30℃ for 24 h. (a) 1% bacterial enrichment cultures from HJ were heat-shocked at 80℃ for 20 min and plating on LB agar plates containing1mM Cd^2+^; (b) 1% bacterial enrichment cultures from LJ were heat-shocked at 80℃ for 20 min and plating on LB agar plates containing1mM Cd^2+^.

**Table S1** Bacterial 16S rRNA gene sequences and OTUs of all samples.

| **Samples** | **Raw sequences no.** | **Final sequences no.** | **OTUS no.** |
| --- | --- | --- | --- |
| LA1 | 34336 | 16997 | 1645 |
| LA2 | 36107 | 17294 | 1641 |
| LA3 | 22863 | 10812 | 1440 |
| LJ1 | 38785 | 20142 | 1870 |
| LJ2 | 39164 | 22616 | 1866 |
| LJ3 | 38235 | 19965 | 1794 |
| LO1 | 35753 | 20891 | 1676 |
| LO2 | 20632 | 11549 | 1476 |
| LO3 | 34168 | 18068 | 1935 |
| HA1 | 40916 | 21300 | 1901 |
| HA2 | 43914 | 22951 | 1969 |
| HA3 | 34758 | 17555 | 1731 |
| HJ1 | 34306 | 17065 | 1982 |
| HJ2 | 38100 | 18256 | 2061 |
| HJ3 | 50017 | 24715 | 2252 |
| HO1 | 36267 | 23059 | 1466 |
| HO2 | 25228 | 16332 | 1187 |
| HO3 | 29728 | 18510 | 1426 |
| **Total** | **633277** | **338077** | **31318** |

*r*^a^ represent the Pearson correlation coefficient between the bacterial relative abundance and Cd concentration in soil, *p*^b^ represent the *p*-value of the bacterial relative abundance difference between the H and L samples in t-test analysis.

**Table S2. Cd-MIC, Cd^2+^ removal rate and 16S rDNA identification for isolated strains**

| Strains | Cd-MIC (mM) ^a^ | Cd^2+^ removal rate (%)^b^ | 16S rDNA identification |
| --- | --- | --- | --- |
| la-1 | 2.00 | 41.38±4.99 | *Burkholderia sp.* |
| la-2 | 3.00 | 18.45±2.30 | *Achromobacter sp.* |
| lj-1 | 4.00 | 31.48±4.06 | *Enterobacter sp.* |
| lj-2 | 3.00 | 13.23±1.22 | *Enterobacter sp.* |
| lj-3 | 3.00 | 14.05±0.27 | *Burkholderia sp.* |
| lo-1 | 3.00 | 9.48±1.93 | *Burkholderia sp.* |
| lo-2 | 3.00 | 7.10±0.97 | *Pandoraea pnomenusa* |
| ha-1 | 5.00 | 81.78±3.64 | *Burkholderia sp.* |
| ha-2 | 4.00 | 6.54±0.72 | *Burkholderia sp.* |
| hj-1 | 3.00 | 8.39±2.96 | *Burkholderia sp.* |
| hj-2 | 5.00 | 79.37±2.12 | *Burkholderia sp.* |
| hj-3 | 3.00 | 14.21±1.79 | *Achromobacter sp.* |
| hj-4 | 4.00 | 22.77±1.36 | *Burkholderia sp.* |
| hj-5 | 4.00 | 8.96±1.30 | *Pseudomonas sp.* |
| ho-1 | 3.00 | 17.43±1.17 | *Burkholderia sp.* |
| ho-2 | 3.00 | 10.53±1.04 | *Burkholderia sp.* |
| ho-3 | 6.00 | 63.05±2.09 | *Burkholderia sp.* |
| *E. coli* BL21 | 2.00 | 9.57±1.34 | */* |

^a^ The Cd-MIC was determined in LB liquid medium into 96-well (12×8) microtiter plates at varying concentrations of Cd^2+^ (0, 1, 2, 3, 4, 5, 6 and 7 mM). ^b^ Cd^2+^ removal rate was measured in LB liquid medium supplementation with 0.1 mM CdCl_2_ and shaken at 200 rpm at 30 °C for 24 h.

**Table S3. Cd-MIC, Cd^2+^ removal rate and 16S rDNA identification for isolated *Bacillus* sp. strains**

| Strains | Cd-MIC (mM) ^a^ | Cd^2+^ removal rate (%)^b^ | 16S rDNA identification | Source (isolated from HJ or LJ) |
| --- | --- | --- | --- | --- |
| 151-4 | 0.6 | 22.18±0.013 | *Uncutrured Bacillus* | HJ |
| 151-5 | 0.8 | 84.78±5.99 | *Bacillus aquimaris* | HJ |
| 151-6 | 0.4 | 90.14±7.50 | *Uncutrured Bacillus* | HJ |
| 151-7 | 0.6 | 57.96±0.086 | *Bacillus vietnamensis* | HJ |
| 151-8 | 0.4 | 19.64±2.35 | *Bacillus aquimaris* | HJ |
| 151-9 | 0.4 | 73.20±0.20 | *Uncutrured Bacillus* | HJ |
| 151-10 | 0.6 | 65.34±6.28 | *Bacillus sp.* | HJ |
| 151-11 | 0.8 | 36.12±4.45 | *Bacillus aquimaris* | HJ |
| 151-12 | 0.6 | 75.34±0.29 | *Bacillus vietnamensis* | HJ |
| 151-13 | 0.8 | 82.82±1.39 | *Bacillus sp.* | HJ |
| 151-15 | 0.6 | 5.29±8.77 | *Bacillus aquimaris* | HJ |
| 151-16 | 0.4 | 69.75±0.28 | *Bacillus aquimaris* | HJ |
| 151-17 | 0.4 | 29.28±1.14 | *Uncutrured Bacillus* | HJ |
| 151-18 | 0.4 | 42.03±2.97 | *Uncutrured Bacillus* | HJ |
| 151-19 | 0.4 | 50.85±2.49 | *Uncutrured Bacillus* | HJ |
| 151-20 | 0.4 | 82.39±0.71 | *Bacillus megaterium* | HJ |
| 151-21 | 0.6 | 81.79±0.65 | *Bacillus sp.* | HJ |
| 151-22 | 0.8 | 25.02±1.16 | *Bacillus aquimaris* | HJ |
| 151-23 | 0.4 | 84.18±0.98 | *Bacillus vietnamensis* | HJ |
| 151-24 | 0.6 | 71.16±6.17 | *Uncutrured Bacillus* | HJ |
| 151-25 | 1.0 | 22.80±3.15 | *Bacillus subtilis* | HJ |
| 152-1 | 0.4 | 15.84±1.71 | *Bacillus amyloliquefaciens* | LJ |
| 152-2 | 0.6 | 17.92±0.99 | *Bacillus amyloliquefaciens* | LJ |
| 152-3 | 0.6 | 7.74±0.95 | *Bacillus amyloliquefaciens* | LJ |
| 152-4 | 0.4 | 37.94±1.90 | *Bacillus amyloliquefaciens* | LJ |

^a^ The Cd-MIC was determined in LB liquid medium into 96-well (12×8) microtiter plates at varying concentrations of Cd^2+^ (0, 1, 2, 3, 4, 5, 6 and 7 mM), the part of data came from our previous study. ^b^Cd^2+^ removal rate was measured in LB liquid medium supplementation with 0.1 mM CdCl_2_ and shaken at 200 rpm at 30 °C for 24 h.
